# Supplementary material for: Tead1a Initiates Transcriptional Priming Through the TEAD1a/YAP‐Notch1‐Spi1/Cebpα Axis to Promote Neutrophil Fate
Source: Adv Sci (Weinh). 2025 Aug 28;12(41):e05441. doi: 10.1002/advs.202505441 (PMC12591127; doi:10.1002/advs.202505441)
Supplement: Supplementary file 4 — Supplementary Dataset [file ADVS-12-e05441-s004.zip › 3-1.zip!/3-1/02-1.cluster/cluster2/Enrich/ego_all.pdf]

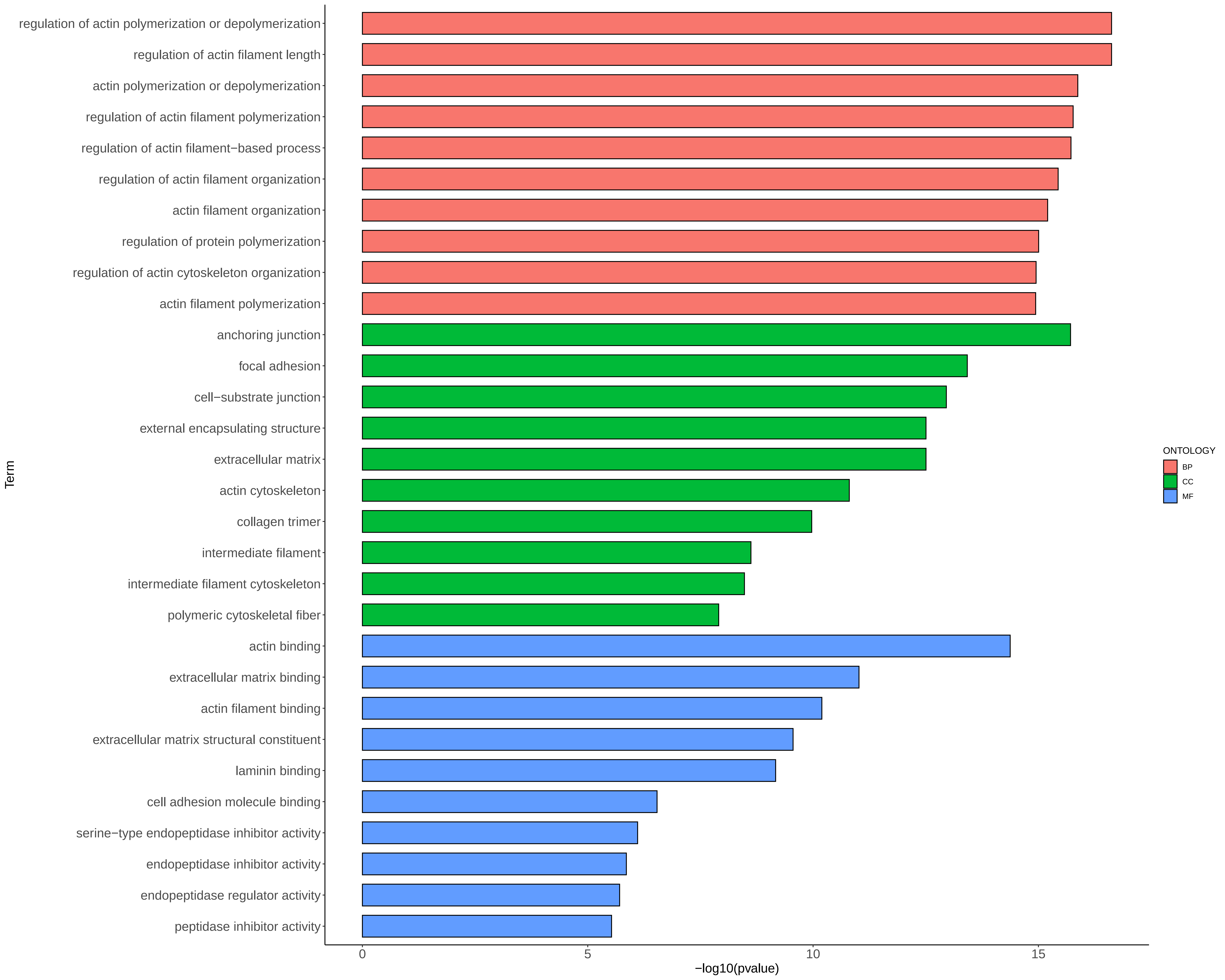

regulation of actin polymerization or depolymerization
regulation of actin filament length
actin polymerization or depolymerization
regulation of actin filament polymerization
regulation of actin filament−based process
regulation of actin filament organization
actin filament organization
regulation of protein polymerization
regulation of actin cytoskeleton organization
actin filament polymerization
anchoring junction
focal adhesion
cell−substrate junction
external encapsulating structure
extracellular matrix
actin cytoskeleton
collagen trimer
intermediate filament
intermediate filament cytoskeleton
polymeric cytoskeletal fiber
actin binding
extracellular matrix binding
actin filament binding
extracellular matrix structural constituent
laminin binding
cell adhesion molecule binding
serine−type endopeptidase inhibitor activity
endopeptidase inhibitor activity
endopeptidase regulator activity
peptidase inhibitor activity
Term
0
5
10
15
−log10(pvalue)
ONTOLOGY
BP
CC
MF
